# Supplementary material for: ChiraKit: an online tool for the analysis of circular dichroism spectroscopy data
Source: Nucleic Acids Res. 2025 Apr 26;53(W1):W158–68. doi: 10.1093/nar/gkaf350 (PMC12230716; doi:10.1093/nar/gkaf350)
Supplement: gkaf350_Supplemental_File [file gkaf350_supplemental_file.pdf]

Supporting Information

ChiraKit, an online tool for the analysis of circular dichroism spectroscopy data

Osvaldo Burastero*[1,2], Nykola C. Jones[3], Lucas A. Defelipe[1,2],
Uroš Zavrtanik[4], San Hadži[4], Søren Vrønning Hoffmann[3], & Maria M. Garcia-Alai*[1,2]

1 European Molecular Biology Laboratory Hamburg, Notkestrasse 85, 22607, Hamburg, Germany
2 Centre for Structural Systems Biology, Notkestrasse 85, 22607 Hamburg, Germany
3 ISA, Department of Physics and Astronomy, Aarhus University, Ny Munkegade 120, 8000, Aarhus, Denmark
4 Department of Physical Chemistry, Faculty of Chemistry and Chemical Technology, University of Ljubljana, Večna pot 113, 1000 Ljubljana, Slovenia

Corresponding authors

Maria M. Garcia Alai (maria.garcia@embl-hamburg.de)
Osvaldo Burastero (oburastero@embl-hamburg.de)

Supplementary Figures

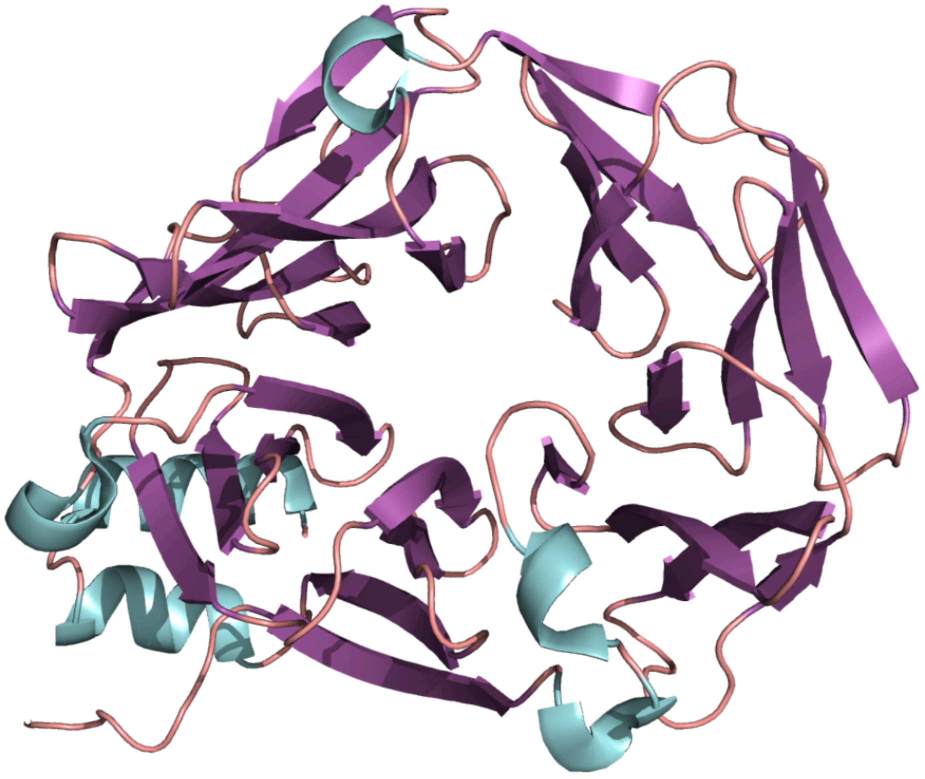

Figure S1. X-ray structure of the human Clathrin Heavy Chain N-Terminal Domain (HsCHC-NTD, PDB code: 9C0Y). The structure is shown in cartoon representation, with alpha helices colored in cyan, beta sheets in purple, and loops in salmon.

Protein - DNA binding affinity

The interaction of berenil with calf thymus DNA was reported by Garbett, Nichola C. *et al.*, 2007 (1). We digitised the data from Figure 1B, which represents the CD signal (in millidegrees) at 385 nm versus DNA concentration, and analysed it using the ‘Custom analysis’ panel. We applied a binding model that assumes DNA does not contribute to the CD signal, and different signal intensities between the bound and free states of berenil. The fitted equilibrium dissociation constant (K_D) was 78 µM, in agreement with the reported one (50 µM) (Figure S1). The function string used to estimate the K_D in the ‘Custom analysis’ panel was:

'0.5*(ABsignal - freeAsignal) * ((10**logTenOfKd+6+Bconc) - sqrt((10**logTenOfKd+6+Bconc)**2 - 4*6*Bconc)) + freeAsignal*6'

It represents the Equation:

$$Y = 0.5(C - A) * (10^{x} + 6 + [DNA] - \sqrt{(10^{x} + 6 + [DNA])^{2}} + 6A$$

where C and A weight, respectively, the complex and free DNA contribution to the signal. The parameter to be fitted is x (10^{x} equals the K_D).

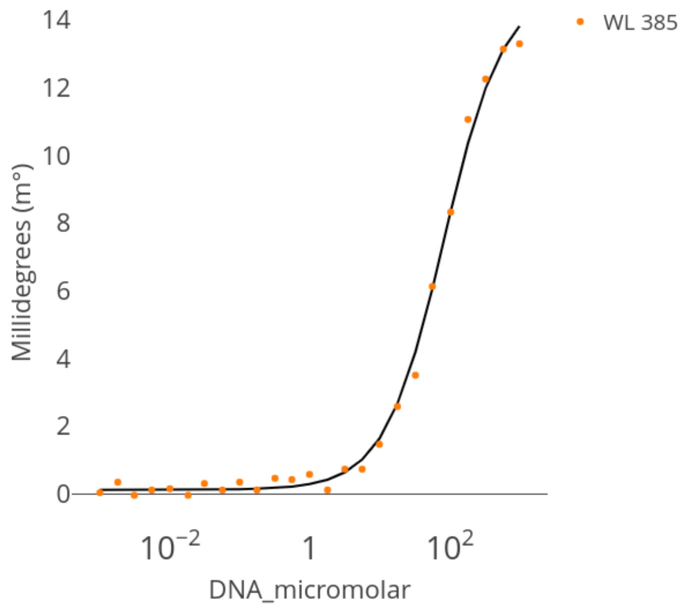

Figure S2. CD intensity (at 385 nm) versus the logarithm of the DNA concentration (orange dots). The data was taken from Garbett, Nichola C. *et al.*, 2007. The fitted curve assuming a one-to-one binding model is shown as a black line.

Two-state unfolding of the Arc dimer

The unfolding reaction of the Arc repressor was explained by a two-state transition from folded dimer to unfolded monomer ($N_2 \rightleftharpoons 2U$) by Bowie and Sauer, 1989 (2). We globally fitted the normalised fluorescence data (digitised from Figure 3a from ref. (2)) yielding parameters similar to those previously reported (Figure S2). We obtained an *m*-value of 2 kcal/mol/M, compared to the published *m*-value of 1.9 kcal/mol/M.

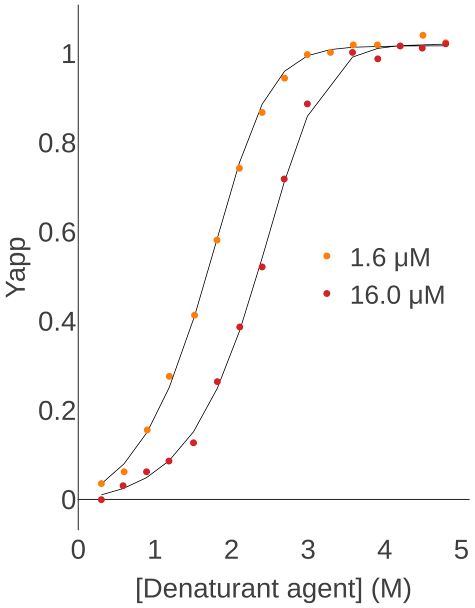

Figure S3. Apparent unfolded fraction of the Arc repressor as a function of urea concentration at two different protein concentrations. Graph created with Chirakit using the

raw data from (2) (red and orange dots). Black lines correspond to ChiraKit based two-state ($N_2 \rightleftharpoons 2U$) global fitting of the data extracted from the Figure in panel A.

Three-state unfolding of the FtsZ dimer

FtsZ, a major protein in bacterial cytokinesis that polymerizes into single filaments, unfolds in urea via a dimeric intermediate ($N_2 \rightleftharpoons I_2 \rightleftharpoons 2U$), as presented by Montecinos-Franjola *et al.*, 2012 (3). The normalised CD data presented in Figure 3A was digitised and globally fitted, obtaining comparable results (Figure S3): 6.4 versus 5.9 kcal/mol/M for the m_1-value, and 3.2 versus 2.4 kcal/mol/M for the m_2-value.

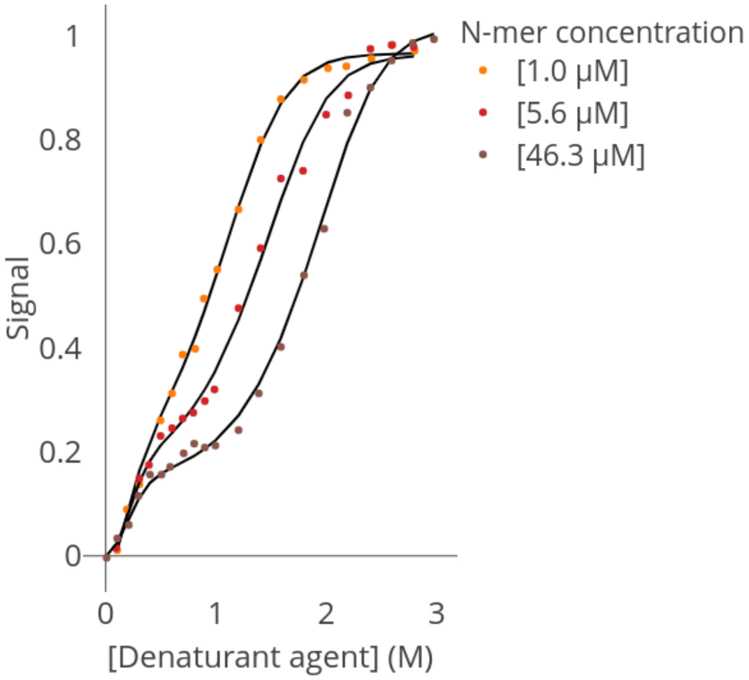

Figure S4. Apparent unfolded fraction of FtsZ as a function of urea concentration at three different protein concentrations (circles: 1 μM, triangles: 5.6 μM, and squares: 46.3 μM). Graph created with Chirakit using the raw data from (3) (red, orange and brown dots). Black lines correspond to ChiraKit based three-state ($N_2 \rightleftharpoons I_2 \rightleftharpoons 2U$) global fitting of the data extracted from the Figure in panel A.

Supplementary Tables

Table S1. Peptide sequences for the study of intrinsically disordered proteins.

Peptide Name	Sequence
c-Myb	Ac-EKRIKELELLLMSTENELKGY-NH2
MLL	Ac-PSDIMDFVLKNTPY-NH2
PaaA2	DPRPAIPHDEVERRMAERFAKMRKERSKQW
RelA	DDRHRIEEKRKRTYETFKSIMKKS

CcdA	RRLRAERWKAENQEGMAEVARFIEMNGSFADENRDW
HigA2	NRDLFAELSSALVEAKQHSEGW
AK32	Ac-$(AAKAA)_6$GY-NH2

Table S2. Comparison between ChiraKit, CDPal, CDToolX, BestSel, CD-NuSS and CalFitter.

**Tool**	ChiraKit	CDPal	CDToolX	BestSel	Dichroweb	CD-NuSS	CalFitter
Online application	✔	-	-	✔	✔	✔	✔
Desktop application	✔	✔	✔	-	-	-	-
Open-source code	✔	✔	-	-	-	-	-
Real-time interactivity	✔	-	✔	-	-	-	-
Tutorials	✔	-	✔	✔	-	✔	✔
Multiple input files	✔	✔	✔	✔	-	-	✔
Displays files metadata	✔	-	-	-	-	-	-
Different input file formats	✔	-	✔	-	✔	-	-
Preprocessing features (e.g., baseline subtraction, average)	✔	-	✔	-	-	-	-
CD units conversion	✔	-	✔	-	-	-	-
SVD/PCA decomposition	✔	-	✔	-	-	-	✔
Rotation or inversion of basis spectra	✔	-	-	-	-	-	-
Estimation of protein secondary structure	✔	-	-	✔	✔	-	-
User-defined reference sets for the secondary structure estimation	✔	-	✔	-	-	-	-
Secondary structure from PDB files	✔	-	-	✔	-	-	-
Estimation of nucleic acid secondary structures	-	-	-	-	-	✔	-

Fitting of chemical/thermal reversible unfolding models for monomers and homodimers	✓	✓	-	-	-	-	✓*
Simulation of chemical reversible unfolding models for monomers and homodimers	-	✓	-	-	-	-	-
Fitting of chemical/thermal reversible unfolding models for homotrimers and homotetramers	✓	-	-	-	-	-	-
Fitting of thermal reversible unfolding models for peptides (helix/coil only)	✓	-	-	-	-	-	-
Fitting of thermal irreversible unfolding models for monomers	✓	-	-	-	-	-	✓
Global fitting of unfolding models at multiple wavelengths or SVD/PCA coefficients	✓	-	-	-	-	-	✓
Helicity estimation of peptides	✓	-	-	-	-	-	-
L2 normalisation to compare spectra	✓	-	-	-	-	-	-
Euclidean distances between spectra	✓	-	-	-	-	-	-
Fitting of ligand-binding models	✓	-	-	-	-	✓	-
Fold recognition	-	-	-	✓	-	-	-
Option to customize and export Figures in different formats (e.g., .svg, .png)	✓	✓	-	-	-	--	-
Combines data from	-	-	-	-	-	-	✓

different techniques							

*CalFitter does not support chemical unfolding models; however, it also offers four-state models.

Table S3. Comparison of lysozyme's secondary structure composition as determined by the SELCON3 method versus structural data.

**Method**	**Alpha helix**	**Beta sheet**	**Turns**	**Other**
SELCON3*	40	12	15	34
DSSP (PDBid 1dpx)	41.4	6.3	23.4	28.9

*The ‘alpha-regular’ and ‘alpha-distorted’ secondary elements were grouped into the ‘alpha’ category. The ‘beta-regular’ and ‘beta-distorted’ secondary elements were grouped into the ‘beta' category.

Table S4. Comparison of lysozyme's secondary structure composition as determined by the SESCA bayesian method versus structural data.

**Method**	**Alpha helix**	**Beta sheet**	**Coil**
SESCA	37 ± 6	9 ± 9	53 ± 7
DSSP-T (PDBid 1dpx)	41.4	6.2	52.3

Table S5. Fitted parameters of the three-state **reversible** model applied to the chemical unfolding of clathrin heavy chain N-terminal domain (CHC-NTD). The first three SVD coefficients were used for the fitting.

**Parameter**	**Lower fitting bound**	**Fitted value**	**Upper fitting bound**	**Relative error (%)**
M1 (kcal/mol/M)	0.2	3.89	20	148
$D50_1$ (M)	0.75	2.5	4.25	3.1
M2 (kcal/mol/M)	0.2	4.07	20	43.2
$D50_2$ (M)	1	3.88	6	1.8

* Rows marked in red contain parameters with relative errors larger than 95 %.

Table S6. Fitted parameters of the three-state **reversible** model applied to the chemical unfolding of clathrin heavy chain N-terminal domain (CHC-NTD). The CD signal at wavelengths 214, 217, 220, 223, and 220 nm were used for the fitting.

**Parameter**	**Lower fitting bound**	**Fitted value**	**Upper fitting bound**	**Relative error (%)**
M1 (kcal/mol/M)	0.2	4.72	20	163
$D50_1$ (M)	0.75	2.49	4.25	1.7

M2 (kcal/mol/M)	0.2	4.01	20	27.1
$D50_2$ (M)	1	3.88	6	1.2

* Rows marked in red contain parameters with relative errors larger than 95 %.

Table S7. Helix contents of intrinsically disordered peptides with helix binding motifs. From the measured CD signals [θ] we estimated fractional peptide helicity f_H using the ensemble model (4) integrated in ChiraKit (fifth column). This is compared to the traditional approach to estimate f_H (5) (sixth column), which gives large error in case of disordered peptides, but is rather accurate for peptides with high helix propensity (eg. alanine peptide, AK32). LR parameter gives a measure of helix propensity. All data were recorded at 25°C.

Peptide name	$N_{pep.bonds}$	[θ] (deg cm^2 $dmol^{-1}$ pep.bonds $^{-1}$)	LR propagation parameter - *w*	f_h (ensemble)	$f_h = ([\theta] - [\theta]_c) / \Delta[\theta]_{h-c}$	Rel_error (%)
c-Myb	23	-4699.1	1.06	0.21	0.13	58
MLL	15	-5001.6	1.20	0.23	0.16	48
PaaA2	29	-7841	1.08	0.3	0.22	34
RelA	23	-2707.8	1.00	0.14	0.07	100
CcdA	35	-1568.8	0.92	0.1	0.03	210
HigA2	21	-1919.3	0.98	0.12	0.05	162
AK32	33	-22574	1.27	0.7	0.66	6

References

1. Garbett,N.C., Ragazzon,P.A. and Chaires,J.B. (2007) Circular dichroism to determine binding mode and affinity of ligand-DNA interactions. *Nat. Protoc.*, **2**, 3166–3172.

2. Bowie,J.U. and Sauer,R.T. (1989) Equilibrium dissociation and unfolding of the Arc repressor dimer. *Biochemistry*, **28**, 7139–7143.

3. Montecinos-Franjola,F., Ross,J.A., Sánchez,S.A., Brunet,J.E., Lagos,R., Jameson,D.M. and Monasterio,O. (2012) Studies on the dissociation and urea-induced unfolding of FtsZ support the dimer nucleus polymerization mechanism. *Biophys. J.*, **102**, 2176–2185.

4. Zavrtanik,U., Lah,J. and Hadži,S. (2024) Estimation of Peptide Helicity from Circular Dichroism Using the Ensemble Model. *J. Phys. Chem. B*, **128**, 2652–2663.

5. Scholtz,J.M., Qian,H., York,E.J., Stewart,J.M. and Baldwin,R.L. (1991) Parameters of helix-coil transition theory for alanine-based peptides of varying chain lengths in water. *Biopolymers*, **31**, 1463–1470.
